# Factors associated with the use of important human antimicrobials in Japanese small-animal clinics

**DOI:** 10.3389/fvets.2025.1496422

**Published:** 2025-03-25

**Authors:** Kohei Makita, Mao Yoshida, Makoto Ukita, Takeshi Matsuoka, Masato Sakai, Yutaka Tamura

**Affiliations:** ^1^WOAH Collaborating Centre Consortium for Food Safety, School of Veterinary Medicine, Rakuno Gakuen University, Ebetsu, Japan; ^2^Japan Veterinary Medical Association, Tokyo, Japan

**Keywords:** companion animal, important antimicrobials, prudent use, awareness, price, ease of use

## Abstract

**Introduction:**

In Japan, programs to monitor antimicrobial use in companion animals have not been established. To fill this gap, the Japan Veterinary Medical Association has conducted surveys of actual use. The aims of this paper are to clarify the frequency and factors associated with the use of human antimicrobials in Japanese small-animal clinics.

**Methods:**

Antimicrobial usage and awareness surveys were conducted at 260 veterinary clinics between November 2021 and February 2022 using two questionnaires. The annual use of each antimicrobial drug was categorized by frequency, and the median value of each category, with a score of 50 for the choice ≥50, was used to quantify usage frequency. Important antimicrobial drugs for human use were defined as rank I antibiotics of the Food Safety Commission. Knowledge and awareness factors associated with the use of important antimicrobial drugs for human use were analyzed using three approaches. First, the use of important drugs was examined using a generalized linear model (GLM) with binomial errors. Second, a vector generalized linear and additive model with zero-inflated binomial errors was used to evaluate the proportion of important drugs among the annual frequency of use of human antimicrobial drugs. Third, at the drug level, selecting veterinary clinics using important human drugs, univariable GLMs with Poisson errors were used to evaluate the frequency of important human drug use, with the log number of employees as the offset term.

**Results:**

The response rates were 71.2 and 72.3% for the antimicrobial usage and awareness surveys, respectively. All of the facilities used human antimicrobial drugs, and 57.1% (93/163) of facilities used important human antimicrobial drugs. Important human antimicrobial drugs accounted for 21.7% of the frequency of use of human antimicrobial drugs annually (7,342/33,896 times). In terms of the proportion of important human drugs and frequency of important human drug use, the use of important human antimicrobial drugs was low in cases of high awareness of prudent use of antimicrobial drugs and where tests for evidence-based judgment were introduced, but was high when price and ease of use were emphasized.

**Discussion:**

Antibiotic stewardship should be further promoted in Japanese small-animal clinics through educational and information dissemination activities.

## 1 Introduction

Antimicrobial resistance (AMR) is a serious health concern for both humans and animals and requires global and multi-sectoral collaborations to address. Companion animals such as dogs and cats live closely with humans and may serve as carries for bacteria exhibiting AMR (1). In 2016, the Japanese National Action Plan on AMR for the years 2016–2020 suggested that surveillance and monitoring systems for AMR in companion animals should be strengthened (2). Based on this recommendation, AMR bacteria in companion animals were included in the Japanese Veterinary Antimicrobial Resistance Monitoring System in 2017 (3). In 2017, monitoring of AMR bacteria in companion animals focused on diseased animals, but in 2018, monitoring was initiated in healthy dogs and cats (4). The second phase of the Japanese National Action Plan on AMR for the years 2023–2027 stated that the survey to estimate the amount of human antimicrobial drugs used to treat companion animals would be continued (5). The estimated amounts (in metric tons) of animal and human antimicrobial drugs sold for the treatment of companion animals between 2013 and 2018 have been reported elsewhere (4). However, these estimates did not include imported drugs which are not approved for sales in Japan but personally imported by veterinarians (hereafter imported drugs), and no program to monitor actual antimicrobial drug use in companion animal treatment has been established. In the Federation of Asian Veterinary Associations (FAVA) Strategy to tackle AMR 2021–2025, FAVA stated to contribute to regional and national efforts on surveillance and research on AMR, antimicrobial use and residues in animals (6).

In response to this situation, the Japan Veterinary Medical Association (JVMA), a member of the FAVA, conducted a survey on the actual use of animal, human, and imported antimicrobial drugs at small-animal clinics in 2018 using a structured questionnaire. The results revealed that 61.8, 12.6, and 1.5% of antibacterial drugs used in companion animal clinics were human drugs, important human drugs, and imported drugs, respectively. Furthermore, recently established clinics directed by middle-aged and older individuals used higher proportions of important human drugs to treat animals (7). That study quantified the amounts of antibiotics used in bulk powder weight; however, such aggregated amount data do not provide any information regarding frequency of use, such as the veterinary defined daily dose (8). Moreover, as the questionnaire used in the 2018 survey did not assess knowledge and awareness of AMR, why some veterinarians used important human drugs in large amounts remained unanswered (7).

Currently, the World Health Organization classifies five types of antimicrobials as Highest Priority Critically Important Antimicrobials for Human Medicine (HPCIA): third-generation or later cephalosporins, glycopeptides, macrolides and ketolides, polymyxins, and quinolones (9). The use of such important drugs for human medicine in companion animals may result in the emergence and spread of resistant bacteria against such drugs, and as a consequence, increased failure of treatment in humans against such AMR bacteria. The objectives of the present study, therefore, were to further clarify the frequency of human antimicrobial drug use (particularly critically important antimicrobial drugs for humans) and the factors associated with the use of these drugs in Japanese companion animal clinics.

## 2 Materials and methods

### 2.1 Questionnaire surveys

The JVMA developed two questionnaires to investigate the use of human and imported antimicrobial drugs in small-animal clinics in 2021. The first questionnaire was designed to collect detailed information regarding the types, frequency of use, and awareness associated with the use of various antimicrobial drugs (Table 1). As regulations regarding dosage and frequency of administration differ depending on the drug and animal species, these questions addressed specific animal species. The second questionnaire assessed small-animal clinical veterinarians’ knowledge and awareness/attitudes regarding antimicrobial use, countermeasures implemented at the clinic, and microbiological testing, and suggestions on the improvement of veterinary drugs (Table 1).

**Table 1:** Contents of the two questionnaires used in the study.

Category	Question
Questionnaire for the actual use of human and imported antimicrobials	
Description of antimicrobial drug	Trade names, manufacturers, dosages, and usages of human and imported antimicrobial drugs by animal species
Number of cases per year which the antimicrobial drug was used (selection)	10–19, 20–49, 50 or more by animal species
Awareness of antimicrobial use	Reasons for using it, criteria for deciding whether to use it, and points to keep in mind when using it by animal species
Questionnaire for the knowledge and awareness on the use of antimicrobials	
Facility attributes	Location of the clinic, number of years since the clinic opened, management type of clinic, species of animals treated, number of employees
Knowledge of antimicrobials and drug resistance	Knowledge of second-line drugs, sources of information on antimicrobials, Japanese guidelines, AMR National Action Plan, prudent use of antimicrobials, the monitoring survey of bacteria in companion animals, and the survey of the sales volume of antibiotics for human use to veterinary clinics
Awareness of antimicrobial resistant bacteria	Awareness of antimicrobial resistant bacteria, and concern that antimicrobial resistant bacteria will make treatment in veterinary and human medicine more difficult
Antimicrobial use	Common diseases, reasons for choosing antimicrobials, antimicrobials used on a daily basis, use of medicines not approved for veterinary use in Japan
Use of available veterinary antimicrobials	The ingredients of antimicrobial drugs for human use and the reasons for their use, the basis for dosage and administration when using drugs for human use, and precautions for using antimicrobial drugs
Antimicrobial resistance measures	Education and training in the animal clinic, countermeasures against drug resistance in the clinic, explanations to owners when prescribing antibiotics, prevention of animal clinic-acquired infections, and application of the findings on the monitoring surveys of bacteria in companion animals to veterinary practice
Microbiological testing	Where and when microbiological tests should be performed; whether and why identification of isolated bacteria is necessary; whether and why Gram staining should be performed on isolated bacteria
Suggestions on the improvement of veterinary drugs	Desired dosage forms for veterinary antimicrobials, and antimicrobials that are not available for veterinary use but are considered necessary

A cross-sectional study was designed to cover the entirety of Japan. The sampling framework of small-animal clinics followed the 2021 monitoring survey of AMR bacteria in healthy companion animals conducted by the Japanese Ministry of Agriculture, Forestry and Fisheries (MAFF). Specifically, the total sample size was set at 260, slightly above the sample size of 250 used in the MAFF survey, in consideration of response failure. The survey was proportionally allocated to all prefectures according to the number of small-animal clinics registered by the government. The ethics of the study was evaluated and approved at the sixth AMR Measures Promotion Review Committee of the JVMA on September 29^th^, 2021. Each local veterinary medical association sent requests to cooperate with the survey to the clinics in the prefecture.

A postal survey was conducted in 45 of 47 prefectures, excluding Tokyo and Hokkaido, and the responses were sent to the JVMA between November 29, 2021, and January 10, 2022. For clinics that had not responded by January 10, 2022, an email was sent on January 26 with the URL for the request for online responses by February 18, 2022. In Tokyo Prefecture, an online survey was conducted. In Hokkaido Prefecture, the Hokkaido Veterinary Medical Association Secretariat compiled the responses independently and submitted them to the JVMA.

### 2.2 Statistical analysis

The survey results were digitized using Microsoft Excel (Redmond, WA, USA) and analyzed using descriptive statistic tests. The classification of antimicrobial agents followed the Kyoto Encyclopedia of Gene and Genomes Medicus Drug Search (10). Important antimicrobial drugs for human use were defined as rank I (extremely important) drugs in “Ranking the importance of the antibacterial substances against bacteria that affect human health through food,” published by the Cabinet Office of Japan, Food Safety Commission (11). Ranking questions were weighted in order to generate scores. Regarding annual frequency of antimicrobial use for each drug by animal species, median values of the choices (10–19, 20–49), and 50 for the choice ≥50, were used for quantification purposes. Descriptive statistics were performed to characterize the use of important human drugs in survey clinics.

Knowledge and awareness factors associated with the veterinary use of important antimicrobial drugs for human use were analyzed using three approaches. First, a generalized linear model (GLM) with binomial errors was employed, selecting the binary response for the use of important drugs as the outcome variable and attributes, knowledge, and awareness at the clinic level as explanatory variables. Univariable analyses were performed, and after selecting variables exhibiting *p* < 0.2, multivariable analyses were performed.

Second, a vector generalized linear and additive model with zero-inflated binomial errors was used to evaluate the proportion of important drugs among the annual frequency of human antimicrobial drugs used at the clinic level, using the R package VGAM (12). Variables exhibiting *p* < 0.2 in the univariate analysis were selected, and in the multivariable analysis, selection was performed using a stepwise method. The final model was selected using the Akaike Information Criterion.

Third, at the drug level, selecting veterinary clinics using important human drugs, univariate GLMs with Poisson errors were used to evaluate the frequency of use of important human drugs for all indicated animal species, with the log number of employees as the offset term. The number of cases of annual use of each important antimicrobial drug was analyzed without distinguishing between different antibiotics, and no multivariable analysis was performed. All analyses used the statistical software R, version 4.3.0 (13).

## 3 Results

### 3.1 Description of responding clinics

**Figure 1:**
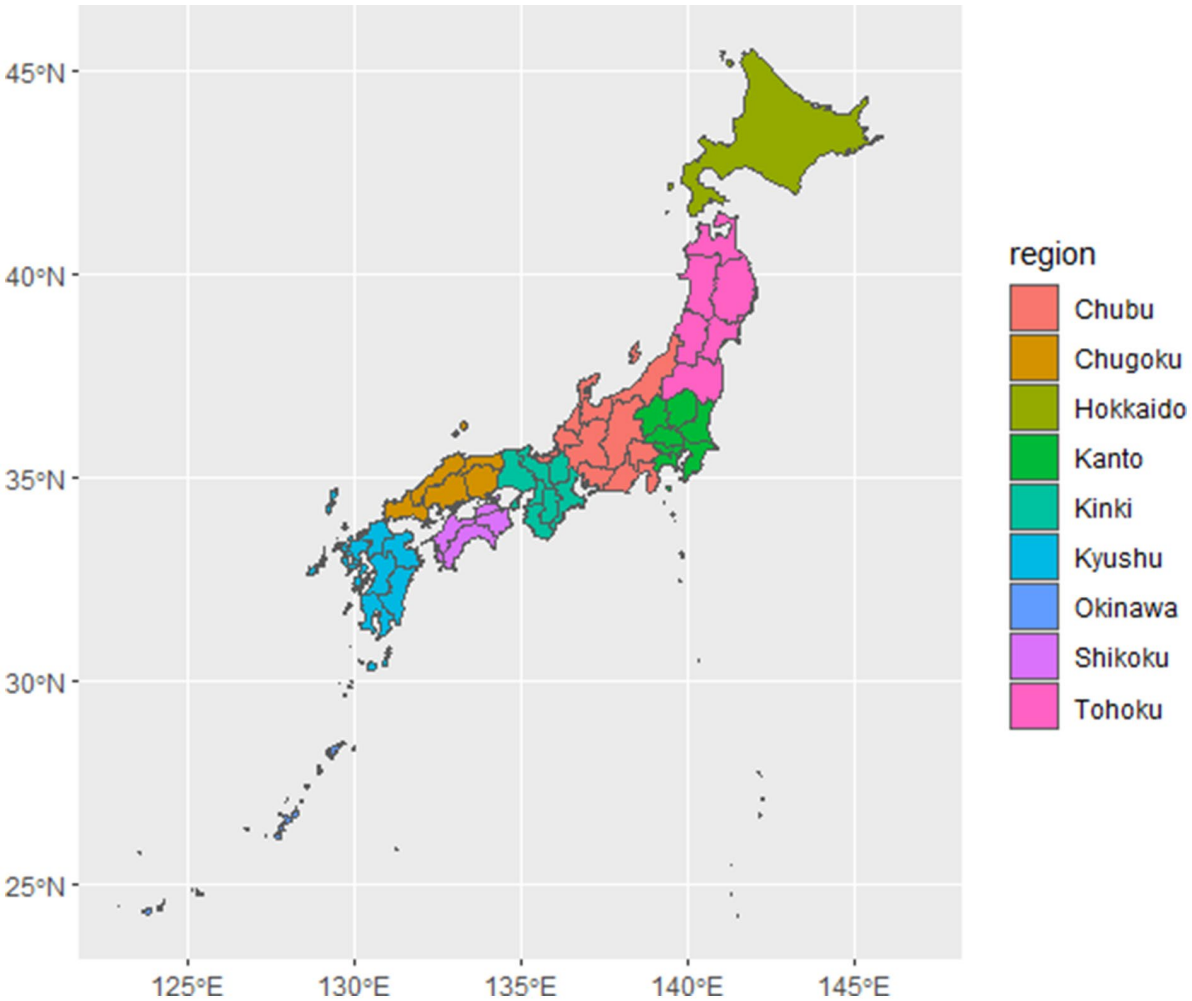
A map showing the locations of regions of Japan.

The response rates for the survey of antimicrobial use and survey of knowledge and awareness were 71.2% (180/260 facilities) and 72.3% (188/260), respectively (Table 2). The response rates by region were slightly lower in the Hokkaido-Tohoku (56.0% for usage and 64.0% for awareness), Kanto (58.3% for usage and 62.1% for awareness), and Chugoku (69.2% for usage and 61.5% for awareness; a map is shown in Figure 1) regions but still generally high. Even among the clinics that responded, some questions were not answered.

**Table 2:** Survey responses and response rates by region.

Region	Number of allocated facilities	Usage survey		Awareness survey	
		Number of responses	Response rate (%)	Number of responses	Response rate (%)
Hokkaido and Tohoku	25	14	56.0	16	64.0
Kanto	103	60	58.3	64	62.1
Chubu	41	36	87.8	36	87.8
Kinki	48	37	77.1	37	77.1
Chugoku	13	9	69.2	8	61.5
Shikoku	6	6	100.0	5	83.3
Kyushu-Okinawa	24	23	95.8	22	91.7
Total	260	185	71.2	188	72.3

The mean number of employees per clinic was 8.1 (median: 6, quartiles: 4.0–9.0, range: 1–3.7). The average number of years since opening was 21.3 (median: 2.1, quartiles: 11.0–28.8, range: 0–68). In terms of form of management, 65.1% (110/169 facilities) were corporations, and 34.9% (5.9/169) were managed by individuals. All clinics provided primary veterinary care, and of the 169 responding clinics, 25 (14.8%) also provided secondary veterinary care. Four clinics (2.4%) provided night-time care, and no clinics provided house calls.

Regarding target animal species, all 170 facilities that responded to the surveys handled dogs, in addition to which 168 (98.8%) treated cats, 58 (34.1%) small birds, 89 (52.4%) rabbits, 79 (46.5%) hamsters, guinea pigs, and ferrets, 11 (6.5%) reptiles and amphibians, and 11 (6.5%) ornamental fish.

### 3.2 Characteristics of human and imported antimicrobial drugs used

Of the total responses according to antimicrobial drug and animal species, antifungals accounted for 2.5% (956/38,458), antivirals for 0.2% (84/38,458), antiparasitic drugs for 7.6% (2,916/38,458), and in total 10.3% (3,956/38,458) of responses indicated agents other than antibacterial drugs. Of the total number of antibacterial drugs used (34,502), the proportion of antibacterial drugs that were indicated as imported and confirmed to be manufactured overseas was 1.8% (606), and the proportion specified as human antibacterial drugs and actually manufactured domestically for human use in Japan was 98.2% (33,896/34,502).

Here after, the results only on the antibiotics approved for human use are described. Of the 932 responses regarding the administration route of human antibiotics for all animal species, the most common indicated route was oral administration (69.8%, 651), followed by intravenous (9.1%, 85), eye drop (8.2%, 76), and subcutaneous (4.2%, 39). There was a total of 1,009 responses for the antibiotics for all animal species from 170 facilities, with use of antibiotics in ≥50 cases the most common response (41.7%, 414 responses), followed by 10–19 cases (32.9%, 326 responses), and 20–49 cases (25.5%, 253 responses).

Table 3 shows the rankings for antibiotic classes among antibiotics for human use according to the estimated number of animal treatment cases per year. Among the important antimicrobial drugs for human use, macrolides with a 14- or 15-membered ring structure ranked 4th (3,092 cases), fluoroquinolones 6th (1,978 cases), third-generation cephalosporins 7th (1,834 cases), and carbapenems 14th (348 cases). The total number of these cases, 7,342, accounted for 21.7% of the total number of animal treatment cases that used human antibiotics per year.

**Table 3:** Ranking of antibiotic classes among antimicrobials for human use according to the number of animal treatment cases per year.

Antibiotic class name	Rank	Number of cases of use	Percentage
Penicillins	1	7,432	21.93%
Tetracyclines	2	6,754	19.93%
First-generation cephalosporins	3	4,602	13.58%
**14- and 15-membered ring macrolides**	**4**	**3,092**	**9.12%**
Aminoglycosides	5	2,020	5.96%
**Fluoroquinolones**	**6**	**1,978**	**5.84%**
**Third-generation cephalosporins**	**7**	**1,834**	**5.41%**
Amphenicols	8	1,126	3.32%
Lincosamides	9	1,110	3.27%
Fosfomycin	10	1,026	3.03%
Sulfamethoxazole-trimethoprim	11	850	2.51%
Penems	12	476	1.40%
Second-generation cephalosporins	13	396	1.17%
**Carbapenems**	**14**	**348**	**1.03%**
Sulfamethoxazole	15	246	0.73%
New quinolones	16	196	0.58%
erythromycin	17	132	0.39%
Steroids	18	82	0.24%
Amphotericin B	19	78	0.23%
**4th generation cephalosporins**	**20**	**48**	**0.14%**
16-membered ring macrolides	21	28	0.08%
**Antituberculosis drugs**	**22**	**28**	**0.08%**
**Glycopeptides**	**23**	**14**	**0.04%**
Total		33, 896	1 00%

^*^Important antibiotics for human use are indicated in bold.

The average number of cases per year in which human antibiotics were used per facility was 178.8 (median: 149, range: 14–1,186). Human antibiotics were used in all surveyed clinics, but the proportion of clinics using important human antimicrobial drugs was 57.1% (93/163 clinics). Out of the human antimicrobial drug uses with indication of administration routes (31,240), oral (74.2%, 23,184), intravenous (9.3%, 2,894), eye (9.1%, 2,858), and subcutaneous (4.4%, 1,362) administrations accounted for 97.0%. Out of these routes, the proportion of the drugs used in the route among important human drugs usage was significantly higher than that among the other human drugs in intravenous (11.3%, 736/6,490, vs. 8.7%, 2,158/24,750, *x*^2^ = 41.7, df = 1, *p* < 0.001) and eye (24.2%, 1,570/6,490, vs. 5.2%, 1,288/24,750) routes, whereas the proportion among important human drugs usage was significantly lower than that among the other human drugs in oral (58.1%, 3,770/6,490, vs. 78.4%, 19,414/24,750, *x*^2^ = 1,111.7, df = 1, *p* < 0.001), and subcutaneous (2.2%, 140/6,490, vs. 4.9%, 1,222/24,750, *x*^2^ = 94.6, df = 1, *p* < 0.001) routes.

### 3.3 Knowledge and attitude/awareness of AMR

In terms of knowledge regarding first- and second-line drugs, 60.5% of respondents (112/185 clinics) knew the specific ingredients. The most common sources of information regarding antibiotics were journals and textbooks (81.1%, 150/185), followed by academic conferences (41.6%, 77/185) and pharmaceutical companies (34.1%, 63/185). The use of Japanese guidelines for prudent use of antimicrobials in companion animals was limited: 2.2% (4/184) of clinics frequently used these guidelines, whereas 26.1% (48/184) used them occasionally, and 32.1% (59/184) knew about but did not use the guidelines. A total of 23.5% (43/183) and 52.4% (97/185) of clinics responded that they knew about the National AMR Action Plan and the guidelines for prudent use of antibiotics, respectively. Regarding the monitoring survey for bacteria in companion animals conducted by the MAFF, 41.6% of clinics (77/185) knew about it, and 30.1% (52/173) utilized the results of that survey in clinical practice. In addition, 12.4% of clinics (23/185) knew about the survey regarding the sales volume of human antibiotics to veterinary clinics.

The majority of clinics (83.2%, 153/182) reported ineffectiveness in treating bacterial infections and the need to be aware of AMR. Only 1.6% of clinics (3/183) responded that drugs not approved as veterinary medicines in Japan (such as human medicines, or personally imported medicines) should not be used, whereas 21.9% (40/183) responded that these drugs should not be used unless it is unavoidable, and 66.7% (122/183) responded that although the use of these drugs is of concern, it is acceptable to use them at the veterinarian’s discretion. When asked what to be careful about when using antibiotics, the most common answer was effectiveness, at 87.6% (162/185), followed by suppression of resistant bacteria at 58.4% (108/185), safety at 57.3% (106/185), and price at 35.1% (65/185).

Cephalexin and amoxicillin are available as veterinary medicines, and all responding clinics indicated awareness of these drugs. Of 183 responding clinics, 33.9% (62 clinics) used human cephalexin, and the most common reason given was ease of administration and dosage (51.6%, 32/62 clinics), followed by the drug’s low cost (40.3%, 26/62).

In terms of the reason given for using human antibiotics, there were 975 responses for all animal species. Of these 975 responses, the most common reason given was because no veterinary medicines containing the same ingredients are sold in Japan (64.5%, 629), followed by ease of administration (20.3%, 198) and affordability (13.2%, 129).

Regarding the criteria used to decide whether to use human antibiotics, there were 1,214 responses for all animal species. The most common reason given for deciding to use human antibiotics was perceived higher effectiveness, at 53.4% (648/1,214), followed by use only when other antibiotics are not effective (19.2%, 232), when other antibiotics are ineffective and unavoidable (16.6%, 202), and no special awareness, use as needed (7.7%, 94). A few responses indicated the decision to use human drugs was based on drug sensitivity test results (1.5%, 18).

### 3.4 AMR countermeasures practiced

Regarding countermeasures against AMR, 44.5% of clinics (81/182) conducted seminars on antibiotics, and 6.4% (11/182) conducted seminars on prudent use. Infection prevention measures were consciously implemented by 34.1% of clinics (63/185).

When administering antibiotics, only 3.2% (6/185) of the clinics always performed microbiological tests to select the appropriate drug based on understanding of the characteristics and drug sensitivity of the bacteria. A total of 164 facilities (88.6% of 185 clinics) occasionally performed microbiological tests, and of these, 48.8% (80/164) performed such tests when antibiotics were ineffective and 11.0% (18/164) when they suspected infection with AMR bacteria, such as when sick animals were referred from other animal clinics or in poor condition. The most common reasons given by the 11 clinics (5.9%) that never performed microbiological tests were the need to provide prompt treatment (5 clinics) and prohibitive cost of testing (2 clinics). Antimicrobial susceptibility testing of isolated bacteria was always performed in 38.5% of clinics (70/182) and occasionally in 45.1% (84/182).

When prescribing antibiotics, 42.2% of clinics (78/185) explain to animal owners the importance of administering the antibiotics exactly as instructed by the veterinarian, linking this to the issue of AMR, whereas 41.1% (76/185) do not explain the issue of AMR but do explain the importance of administering the antibiotics properly.

### 3.5 Suggestion of the improvement of veterinary antimicrobials

Regarding dosage form, 63.2% of clinics (117/185) preferred tablets, 57.8% (107/185) injection, 55.1% (102/185) chewable or flavored drug, 49.5% (86/185) liquid (i.e., paste), and 29.7% (55/185) powder. Other responses included a preference for having all dosage forms and dosages of antibiotics available to facilitate ease of administration on a case by case basis rather than preferring a specific dosage form and a preference for film-coated tablets that correspond to weight or small, easy-to-crush tablets.

### 3.6 Factors associated with the use of important human antimicrobials in animals

This study used data from 163 clinics that responded to two surveys to measure antimicrobial use and awareness. Univariate analysis based on whether a clinic does or does not use important human drugs showed that clinics aware of the monitoring survey of bacteria in companion animals were significantly less likely to use important human antibiotics (odds ratio [OR] = 0.53, *p* = 0.049, Table 4). However, multivariable analysis showed no significant relationship (Table 5).

**Table 4:** Results of univariable analysis of factors associated with the use of important human antimicrobials (*p* < 0.2).

Item	Odds ratio	95% confidence interval	*p-*value
Empirical antibiotic use	1.58	0.82–3.02	0.171
Drugs that are not approved as veterinary medicines in Japan should not be used, or should not be used except in unavoidable circumstances	0.57	0.28–1.18	0.132
I am aware of the monitoring survey of bacteria in companion animals conducted by the Ministry of Agriculture, Forestry and Fisheries	0.53	0.28–1.00	0.049

**Table 5:** Results of multivariable analysis of factors associated with the use of important human antimicrobials.

Item	Odds ratio	95% confidence interval	*p-*value
Drugs that are not approved as veterinary medicines in Japan should not be used, or should not be used except in unavoidable circumstances	0.60	0.29–1.25	0.171
I am aware of the monitoring survey of bacteria in companion animals conducted by the Ministry of Agriculture, Forestry and Fisheries	0.60	0.31–1.13	0.113

In the analysis of factors associated with the proportion of important human antimicrobial drugs used, a number of knowledge- and awareness-related factors with a *p*-value <0.2 were identified (Table 6). Most of the significant factors identified in the univariable analysis remained in the final multivariable model, with all factors statistically significant (Table 7). The ORs were < 1.0 for the factors indicative of higher knowledge level, such as knowing the specific ingredients included in first- and second-line drugs (OR = 0.83), positive attitude-related factors, such as explaining to owners the importance of administrating antibiotics properly (OR = 0.87), and factors related to prudent use of antimicrobials, such as selecting antibiotics based on the results of susceptibility tests (OR = 0.90), use of cephalexin (OR = 0.89) or amoxicillin (OR = 0.88) in animals, and addressing off-label use of approved veterinary drugs (OR = 0.68). In contrast, the ORs were > 1.0 for factors indicating a potentially careless attitude, such as selecting antibiotics based on route of administration (indicating ease of use, OR = 1.34), considering the cost when using antibiotics (OR = 1.31), not performing Gram staining (OR = 1.25), and no concern that use of antimicrobials in animals could negatively impact human medicine (OR = 1.34).

**Table 6:** Results of univariable analysis of factors associated with the proportion of important antimicrobials among human antibacterial drugs used (*p* < 0.2).

Item	Odds ratio	95% confidence interval	*p-*value
Know the specific ingredients of first- and second-line drugs	0.88	0.82–0.94	<0.001
First- and second-line drug knowledge scores	0.91	0.86–0.96	0.001
Select antibiotics based on route of administration	1.15	1.07–1.23	<0.001
Antibiotics are selected based on the results of antimicrobial sensitivity tests	0.94	0.88–1.01	0.070
Use from first-line drugs	0.61	0.55–0.68	<0.001
Use of effective antibiotics	0.85	0.79–0.91	<0.001
Use from broad-spectrum antibiotics	1.05	0.98–1.12	0.187
Drugs that are not approved as veterinary medicines in Japan should not be used	0.60	0.43–0.83	0.003
Using cephalexin-containing preparations for use in animals	0.87	0.81–0.93	<0.001
Using amoxicillin-containing preparations for use in animals	0.93	0.87–0.10	0.038
Considering the cost when using antibiotics	1.12	1.05–1.20	0.001
No in-house training is provided	1.12	1.04–1.20	0.001
No microbiological testing is performed when administering antibiotics	1.46	1.25–1.72	<0.001
No antimicrobial sensitivity testing of isolated bacteria	1.46	1.29–1.67	<0.001
Gram staining not performed in-house	1.05	0.98–1.12	0.174
There is no concern that AMR bacteria selected through the use of antimicrobials in animals in routine veterinary practice could lead to treatment difficulties in human medicine	1.57	1.37–1.80	<0.001
Address off-label use of approved veterinary drugs	0.80	0.72–0.89	<0.001
Explain to owners the importance of administering antibiotics properly	0.87	0.81–0.93	<0.001
Know about the prudent use of antibiotics	0.90	0.84–0.97	0.003

**Table 7:** Final multivariable model for factors associated with the proportion of important antimicrobials among human antibacterial drugs used.

Item	Odds ratio	95% confidence interval	*p-*value
Know the specific ingredients of first- and second-line drugs	0.83	0.77–0.89	<0.001
Select antibiotics based on route of administration	1.34	1.24–1.44	<0.001
Antibiotics are selected based on the results of antimicrobial sensitivity tests.	0.90	0.83–0.97	0.008
Use from first-line drugs	0.57	0.51–0.64	<0.001
Use of effective antibiotics	0.74	0.68–0.79	<0.001
Drugs that are not approved as veterinary medicines in Japan should not be used	0.43	0.29–0.63	<0.001
Using cephalexin-containing preparations for use in animals	0.89	0.81–0.97	0.012
Using amoxicillin-containing preparations for use in animals	0.88	0.80–0.96	0.006
Considering the cost when using antibiotics	1.31	1.21–1.41	<0.001
Gram staining not performed in-house	1.25	1.16–1.36	<0.001
There is no concern that AMR bacteria selected through the use of antimicrobials in animals in routine veterinary practice could lead to treatment difficulties in human medicine	1.34	1.15–1.57	<0.001
Address off-label use of approved veterinary drugs	0.68	0.59–0.78	<0.001
Explain to owners the importance of administering antibiotics properly	0.87	0.81–0.94	<0.001

Univariate analysis of factors associated with the annual frequency of use of important human antimicrobials showed that the frequency of use of these drugs was significantly higher when the reasons given for use were low cost (estimate = 0.55), ease of use (estimate = 0.12), first choice because of perceived effectiveness (estimate = 0.51), and lack of awareness (estimate = 0.13, Table 8). By contrast, important human antimicrobials were used significantly less frequently in cases in which caution was applied, such as when other drugs were ineffective and no other option was available (estimate = −0.61), or no other veterinary drugs sold in Japan contained the same ingredients (estimate = −0.50, Table 8).

**Table 8:** Results of univariable analysis of factors associated with the annual frequency of use of each important human antimicrobial drug.

Item	Estimated value	Standard error	*p-*value
Reasons for use			
There are no other veterinary medicines sold in Japan that contain the same ingredients	−0.50	0.028	<0.001
Cheap price	0.55	0.033	<0.001
easy to use	0.12	0.044	<0.001
Points to keep in mind when using			
Use only when other antibiotics are ineffective and necessary	−0.61	0.039	<0.001
When efficacy is expected, it is used preferentially as a first-choice drug	0.51	0.028	<0.001
No special awareness, used as needed	0.13	0.045	<0.001

## 4 Discussion

This study aimed to clarify the frequency of veterinary use of human antimicrobial drugs, particularly those antimicrobial drugs that are critically important in human medicine, and the factors associated with their use in companion animal clinics in Japan. The study employed two questionnaire surveys, and the response rate for both questionnaires was >70%. No particular regional bias was observed, so the sampled clinics were considered representative of Japan. Regarding antibiotic use, this study also evaluated imported antibiotics; however, imported antibiotics accounted for only 1.8% of the antibiotics used in the survey clinics, with the remaining 98.2% consisting of human antibiotics sold domestically. Therefore, subsequent analyses focused on human antibiotics, particularly those designated as important rank I antibiotics by the Food Safety Commission.

Of the human antibacterial drugs used in the surveyed animal clinics, 21.7% of uses (7,342 cases) involved important antibacterial drugs, predominantly macrolides with 14- and 15-membered ring structures, fluoroquinolones, and third-generation cephalosporins. The resistance rates of *Enterococcus* spp. among healthy dogs and cats against erythromycin (14-membered ring macrolide) were 28.1 and 29.3%, respectively, and those among diseased dogs and cats were 43.4 and 38.0%, respectively in 2022 in Japan (4). Higher resistance rates of *Staphylococcus pseudintermedius* against erythromycin among diseased dogs and cats were reported in 2022: 77.3 and 89.1%, respectively (4). The resistance rates of *Escherichia coli* against ciprofloxacin (fluoroquinolone) among healthy dogs and cats were 10.5 and 7.1%, and those among diseased dogs and cats were 37.3 and 29.6%, respectively, in 2022 (4). The resistance rates of *S. pseudintermedius* were higher among diseased dogs and cats against ciprofloxacin too: 79.5 and 97.8% in 2022 (4). The resistance rates of *E. coli* against cefotaxime (third-generation cephalosporine) among healthy dogs and cats were 8.8 and 7.1%, respectively, and those among diseased dogs and cats were 25.9 and 24.3%, respectively in 2022 (4). In Europe, the resistance rates of *E. coli* against ciprofloxacin in healthy dogs and cats in Belgium, Italy, and Netherlands were reported to be 7 and 2%, 12 and 28%, and 2 and 0%, respectively, and those against cefotaxime were 0 and 2%, 0 and 6%, and 0 and 0%, respectively, between January 2015 and February 2016 (14). As shown here, the resistance rates of bacteria against the HPCIA were reported to be high among Japanese dogs and cats. Considering a previous report indicating that 4.3 and 61.8% of the antibacterial drugs used in small animal clinics are rank I drugs for human use and human antibacterial drugs, respectively, as based on drug bulk powder weight data from the 2018 survey (7), 7.0% (4.3% divided by 61.8%) of human antibacterial drugs used at that time were rank I drugs for human use. According to the Food Safety Commission list of rank I antibiotics (11), usage can be interpreted in a manner similar to that specified by the HPCIA. The large discrepancy in the use of rank I antibiotics in small animal clinics between the previous 2018 survey and the present study (21.7% based on the number of cases versus 7.0% based on the weight of bulk powder) could be related in part to ease of use (e.g., convenient dosage forms and dosages), which was the most frequently cited reason for using human cephalexin, and in part because it is used more as a lighter weight for a single dose or for treating small animals. Regarding human cephalexin and amoxicillin, several respondents commented that human preparations are useful for reducing drug volume and cost in large dogs (not shown in the results). According to the results, the relative proportions of intravenous and eye routes among important human drug usage were higher than among non-important human drugs, which might support the reasoning.

Second-line veterinary drugs for companion animals used in Japan include HPCIAs such as third-generation or later cephalosporins (excluding carbapenem which has not been approved), macrolides with a 15-membered ring structure, and fluoroquinolones (15). According to the 2018 survey, important human antimicrobials, including both rank I antibiotics for human use (4.3%) and second-line veterinary drugs (8.3%), accounted for 12.6% of total antimicrobial use in Japanese animal clinics based on bulk powder weight (7). In the United Kingdom (UK), critically important antimicrobials were used in 60% of cases of treating infections in dogs and 81% of such cases in cats between 2012 and 2014 (16). A few years later, between 2016 and 2017, 5–6% of antimicrobials used in dogs in the UK were HPCIAs, and the majority of these, 4–5%, were fluoroquinolones (17, 18). Efforts to reduce antimicrobial use have progressed in recent years in the UK and other countries (19). According to the AMR One Health Report of Japan for 2023, the mean estimated annual sales of veterinary antibiotics for dogs and cats between 2013 and 2021 was 7.9 tons, with no clear decreasing trend observed (4). By contrast, the estimated annual sales of human antibiotics to animal clinics decreased from 6.48 tons in 2016 to 4.61 tons in 2021, due primarily to decreases in penicillin sales from 1.93 tons in 2016 to 1.88 tons in 2021, first-generation cephalosporins from 3.08 tons in 2016 to 1.39 tons in 2021, and HPCIAs to a lesser extent (4). To compensate for the reduced usage of human antibiotics, sales of penicillins and first-generation cephalosporins for use in animals increased from 1.57 tons and 2.89 tons in 2016 to 2.29 tons and 3.76 tons in 2021, respectively, whereas sales of HPCIAs for use in animals decreased slightly over the same period (4).

In the present survey, all small animal clinics surveyed treated animals with antimicrobials for human use, but the proportion of clinics that used important antimicrobials for human use was 57.1%, revealing a bias when looking at clinics as a whole. No significant factors were identified in multivariable analysis regarding the characteristics that would distinguish small animal clinics that did not use any important antimicrobials for human use, but univariable analysis identified knowledge of the monitoring survey of bacteria in companion animals as significant. This suggests that >40% of clinics that were highly aware of AMR completely avoided the use of important antimicrobials for human use. Characteristics related to knowledge, awareness, and practice were identified as factors affecting the proportion of important human antibacterial drugs used in animal clinics. Regarding knowledge, the proportion of important antibacterial drugs used was low in clinics that had knowledge regarding the specific ingredients contained in first- and second-line drugs and regarding careful use of antibacterial drugs. With respect to awareness and practice, the proportion of important antibacterial drugs was low at clinics with high awareness of issues related to antimicrobial drug use and that engaged in beneficial habits, such as selecting antibacterial drugs based on antibacterial sensitivity test results, using first-line drugs first, careful use (e.g., making sure to use effective antibacterial drugs), preferential use of veterinary drugs, awareness of the importance of selecting antibacterial drugs based on evidence obtained from microbiological tests, awareness of the risk that using antibacterial drugs in animals in daily veterinary practice may affect human medical care, and a sense of duty to explain the risk of antibacterial resistance to owners. Similar results were obtained in analyses of each important human antibacterial drug, with the frequency of use of important human antibacterial drugs found to be low at clinics exhibiting awareness of careful use practices. Conversely, the frequency of use of these drugs was high at clinics in which priority was placed on price and ease of use.

Antibiotic stewardship is defined as responsible medical judgment and actions to ensure that antimicrobial drugs are available and effective, with a focus on human, animal, and environmental health (20). To promote antimicrobial stewardship in veterinary medicine, various countries have employed evidence-based guidelines for responsible antimicrobial use (15, 21) and benchmarking (19, 22). For example, benchmarking in the Netherlands utilized the Defined Daily Doses for Animals (22), whereas the proportion of HPCIA use per consultation was employed in the UK (19). In addition to these approaches, educational materials for clinics have been created in Japan (22), and educational and follow-up programs have been implemented in the UK (19). Also in the UK, multiple, continuous benchmarking reduced the use of HPCIAs by 23.5% in dogs and 39.0% in cats over a 6-month period (19). The present study revealed that acquiring knowledge regarding veterinary antibiotics and an awareness of and willingness to engage in careful use practices can reduce the use of antibiotics, especially those important for human. Therefore, it is expected that antibiotic stewardship will be promoted in Japan as well through continued educational and information dissemination activities, as well as by the development and dissemination of benchmarking programs by relevant organizations. Antibiotic stewardship in veterinary medicine is not limited to the use of antibiotics in animal clinics; it is also expanding to educational activities for pet owners (23). Antibiotic stewardship for pet owners is the responsibility of veterinarians, and it has been reported that building trust with pet owners, joint decision-making, and clear communication fosters careful use and thorough understanding of the risks associated with antibiotic use, including side effects (24).

The present survey has several limitations. Although the objectives of this survey—understand the actual use of imported and important human antimicrobial drugs in small animal clinics and the factors associated with the use of important human antimicrobials—were achieved, the proportion of HPCIA use per number of consultations at each clinic remains unknown. Because survey questions regarding frequency of use were categorized for each drug, the actual frequency of use could not be determined precisely. Biological sampling was not conducted in the clinics which responded the questionnaire, and analysis for the association between antimicrobial use and AMR was not conducted. In addition, monitoring changes in the use of antimicrobials for animals in Japan, especially HPCIAs, could be difficult. In the UK, the Royal Veterinary College’s Vet Compass (25) and the University of Liverpool’s SAVSNET (26) each collect electronic veterinary medical record information from hundreds of primary small animal clinics, making it possible to monitor changes in use and evaluate hypotheses based on the data. In Japan, it is necessary to consider introducing such a system in order to further promote antimicrobial stewardship in the field of small animal medicine and improve animal welfare, not just in terms of AMR bacteria.

## Data Availability

The raw data supporting the conclusions of this article will be made available by the authors, without undue reservation.
